# Volumetric predictors for shunt-dependency in pediatric posterior fossa tumors

**DOI:** 10.1038/s41598-025-06825-w

**Published:** 2025-06-20

**Authors:** Florian Wilhelmy, Erdem Güresir, Johannes Wach, Martin Vychopen, Ulf Nestler, Gordian Prasse, Lisa Haddad, Johannes Kasper, Tim Wende

**Affiliations:** 1 Department of Neurosurgery, University Hospital Leipzig, Leipzig, Germany; 2 Division of Neuroradiology, University Hospital Leipzig, Leipzig, Germany

**Keywords:** Posterior fossa tumors, Pediatric hydrocephalus, Radiomics, Volumetry, Neuroscience, Surgical oncology

## Abstract

Posterior fossa (PF) tumors are the most common neoplastic entity in pediatric neurosurgery. Children suffering from PF tumors regularly present with hydrocephalus and CSF diversion is a crucial point of treatment. There is an ongoing debate about external ventricular drainage (EVD) management before surgery and its influence on ongoing hydrocephalus treatment afterwards. Beyond onco-surgical aspects, the prevention of shunt-dependency is an important goal in posterior fossa surgery. Various predictors for shunt-dependency after posterior fossa surgery in children have been suggested. Because these predictors may only apply to small subsets of children, and their reliability has been questioned, we evaluated a straightforward, potentially automated, and unbiased method for shunt prediction. In this retrospective radiomic study we analyzed 60 pediatric patients with posterior fossa tumors. Exclusion criteria were age under two years, missing MRI data, tumor location non-exclusive to the PF, traumatic brain injury and less than 6 months follow-up. Ultimately, 36 children met the inclusion criteria. We performed a volumetric assessment of various skull and brain compartments before and after surgery focused on ventricle-brain ratio (VBR). We dichotomized for potential predictors and performed ROC analyses. We evaluated the prognostic parameters for shunt dependency, including supratentorial transependymal edema and VBR, as well as pre- and postoperative radiomic measurements as early prognostic tools. The cutoff in ventricle volume for CSF diversion was 60.9 ml (AUC 0.788, *p* = 0.001). The radiomic-based prediction of shunt dependency with VBR-scoring showed an AUC of 0.783. Postoperative reduction in ventricle size, depicted by the deltaVBR scoring, showed an AUC of 0.719 in predicting shunt-free survival. Perioperative CSF diversion did correlate with postoperative persistent HCP, whereas the odd’s ratio for shunting was decreased, but not significantly lower, when CSF diversion was undertaken perioperatively (AUC = 0.618, OR = 0.273, CI = 0.029–2.577). Ventricle-brain ratio may be a potential predictor for the necessity of CSF diversion. In our cohort, radiomic predictors performed better than hydrocephalus categorization, modified Canadian Preoperative Prediction Rule for Hydrocephalus (mCPPRH) or transependymal edema alone. VBR pre- and deltaVBR postoperatively may be potential tools to predict the need for shunting in pediatric posterior fossa tumor patients. The decision for pre- or intraoperative CSF diversion showed no correlation and no influence on persistent hydrocephalus.

## Introduction

Children’s nervous system neoplasms are most commonly located in the posterior fossa (PF)^1^. The occlusion of cerebrospinal fluid (CSF) pathways leads to hydrocephalic configuration and/or related symptoms in 60–70 of those children^2^. The debate about perioperative management of hydrocephalus is ongoing^3^ and focuses on determining factors for persistent requirement of hydrocephalus treatment. Not only the patients’ medical history^4^ but also the mode of CSF diversion, if and when to divert, has been extensively examined and discussed^5,6^. It remains unclear, whether perioperative CSF diversion via endoscopic third ventriculocisterostomy (ETV) or pre- and intraoperative external ventricular drainage (EVD) is resulting in persistent shunt-dependency, or if it’s merely a consequence of preexisting, unchangeable factors^7^. Furthermore, tools and scores have been proposed to stratify the risk for persistent CSF diversion^8^. However, an accurate prediction of the individual need for shunt placement remains challenging.

New diagnostic tools for hydrocephalus in children with PF tumors include radiomics. Due to recent technical developments facilitating and accelerating segmentation of anatomical brain structures, radiomic features have become a common tool in hydrocephalus diagnostics, risk prediction, therapy evaluation and pathophysiological understanding^9,10^, especially in the field of pediatric posterior fossa tumors^11^. Radiomics are based on multiparametric imaging data and advanced mathematical analyses and can therefore quantify imaging features beyond visual perception.

In this study we investigated easy-to-access prognostic radiomic features for persistent shunt-dependency after PF tumor resection. We focused on potentially automatable pipelines, with an output of definitive characteristics, that facilitate shunt prediction. While machine learning was not used in this study, automated ventricle volumetrics are the first step for fully computable processes. Furthermore we examined the influence of perioperative CSF diversion based on these radiomic predictors, to rule out a causal association between the two. This allows for a better understanding of hydrocephalic configurations and enables optimization of treatment strategies for children with PF tumors.

## Methods

We conducted a single-center, retrospective study on basic volumetric data and shunt dependency in children with posterior fossa neoplasms. We searched our electronic patient database for ICD-10 codes of posterior fossa neoplasms with corresponding operative procedures (suboccipital craniotomies) in children, that underwent surgical resection between 2012 and 2024. Exclusion criteria were age < 2 years, as a significantly higher shunt-rate in younger children has been described before^12^. For better comparability, we followed the example of excluding very young children. We excluded lesions falsely assigned to posterior fossa ICD-10 codes or lesions exceeding the posterior fossa, respectively (e.g. pontine glioma, pinealoblastoma and others). Patients with incomplete MRI-datasets were excluded.

We assessed clinical data such as age at time of surgery, tumor entity and known preoperative markers for hydrocephalus, such as papilledema. Radiologically suggested diagnosis and transependymal, supratentorial periventricular edema were assessed for modified Canadian Preoperative Prediction Rule for Hydrocephalus (mCPRRH) stratification^13^. Milestones for course of treatment included pre- and perioperative CSF diversion (external ventricular drainage (EVD)/endoscopic third-ventriculostomy (ETV), shunt implantation and extent of resection (biopsy, subtotal, gross total). The indication for preoperative CSF diversion was based on clinical presentation, e.g. acute hydrocephalic symptoms, by the senior pyhsician on duty. Radiographic assessment included cerebrum, cerebellum, posterior fossa volume, brain stem and ventricular system as well as tumor and cystic tumor volume, if present. If an EVD was inserted, the drainage regimen was continuous. All data was collected and curated by a board certified neurosurgeon (FW); radiological data was assessed and reviewed by a board certified neuroradiologist (GP). Shunting decisions were made at the discretion of the attending neurosurgeon and were based foremost on EVD output. If no EVD was placed, or if it was already removed, neurological symptoms like headaches, dizziness, emesis, or persisting CSF fistulas provided the basis for shunting decision.

**Fig. 1:**
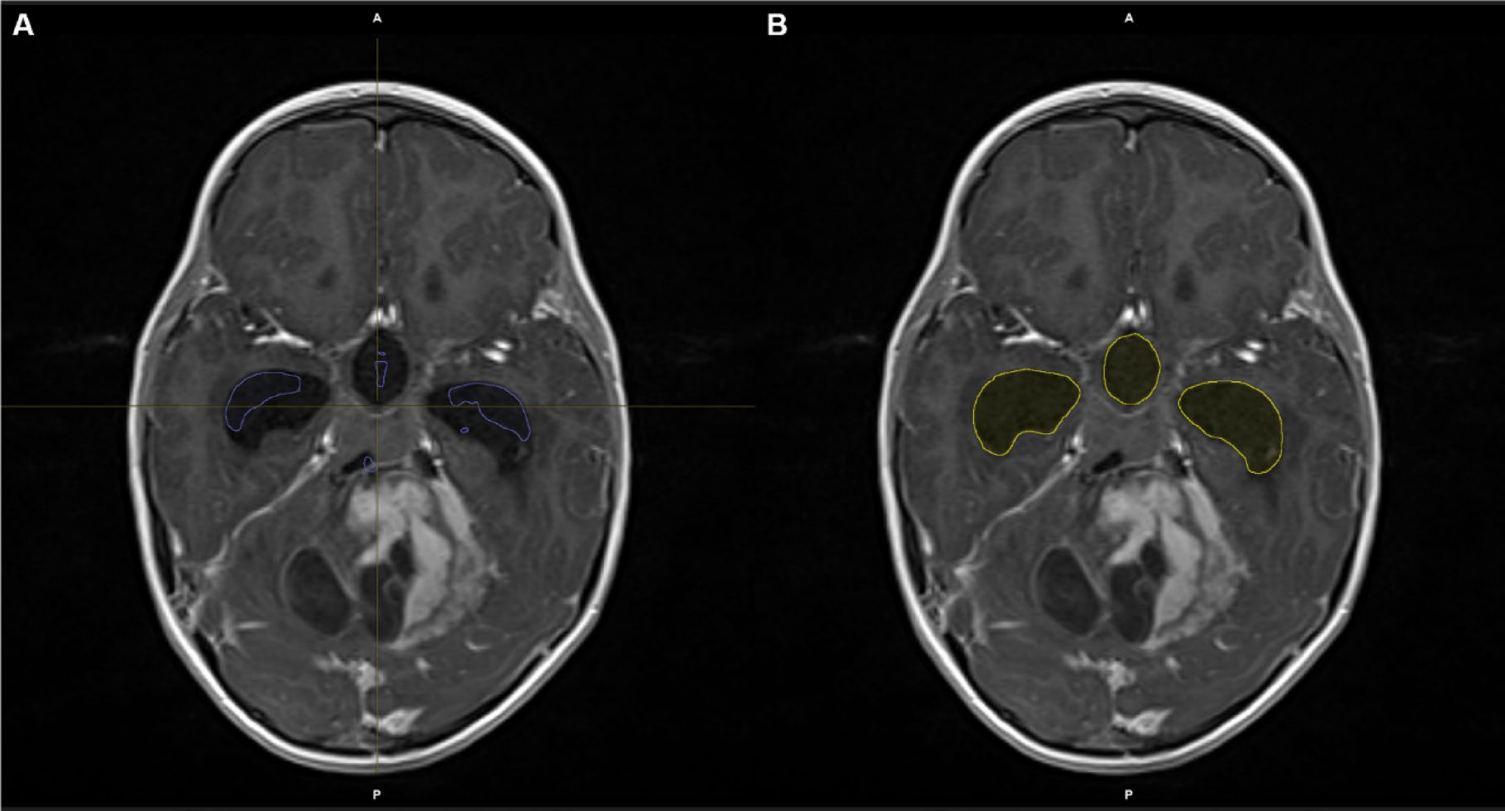
**A**: Automatic Segmentation with Brainlab Elements. Especially in children with severely altered anatomy, automated volumetry can be inaccurate. **B**: In these cases, manual corrections have to be made, in this case with the “smart brush” tool.

Volumetrics were performed using Brainlab Elements (Brainlab AG, München, Germany) using the anatomical automated segmentation and was then reviewed by a neurosurgeon (FW) and a neuroradiologist (GP) in consensus. The automated anatomical segmentation came to its limits in complex pathologies. In these cases, segmentation was corrected manually as shown below (s. Figure 1). We used the “smart brush” tool for slice-by-slice segmentation by hand.

We dichotomized into shunt-dependent and non shunt-dependent children, which was defined by VP-shunt placement during the first 6 months after surgery. Clinical and radiological follow-up data was available for at least 6 month in all children due to oncological aftercare and MRI surveillance. Follow-up was available in our data set for 6 months in all patients, while 3 patients dropped out afterwards. The time-frame was chosen in respect of regular follow-ups in oncological patients, which is 3 and 6 months, respectively. This time frame has been used before elsewhere^14^, while most studies do not state surveillance or shunt placement time-frames. For late onset hydrocephalus, especially later then 6 months, tumor recurrence, radio- and chemotherapy can not be ruled out as influential. Significant differences in between groups were screened as potential predictors. We included ventricle to whole brain (cerebrum, posterior fossa and brainstem combined) volume (VBR) as well as the change in VBR after surgery (deltaVBR) as possible pre- and postoperative shunt predictors via one-sided t-test. We calculated ROC-curves and determined Youden’s index for dichotomization of risk groups. Odd’s ratios were calculated for various predictors. Statisics were performed with SPSS software (IBM, Armonk, USA).

All analyses were carried out according to the ethics committee of the medical faculty, Leipzig University, approval (Az 330–13–18112013) and according to relevant guidelines. The need for informed consent was waived by the ethics committee of the medical faculty, Leipzig University, due to the retrospective nature of the study.

## Results

**Fig. 2:**
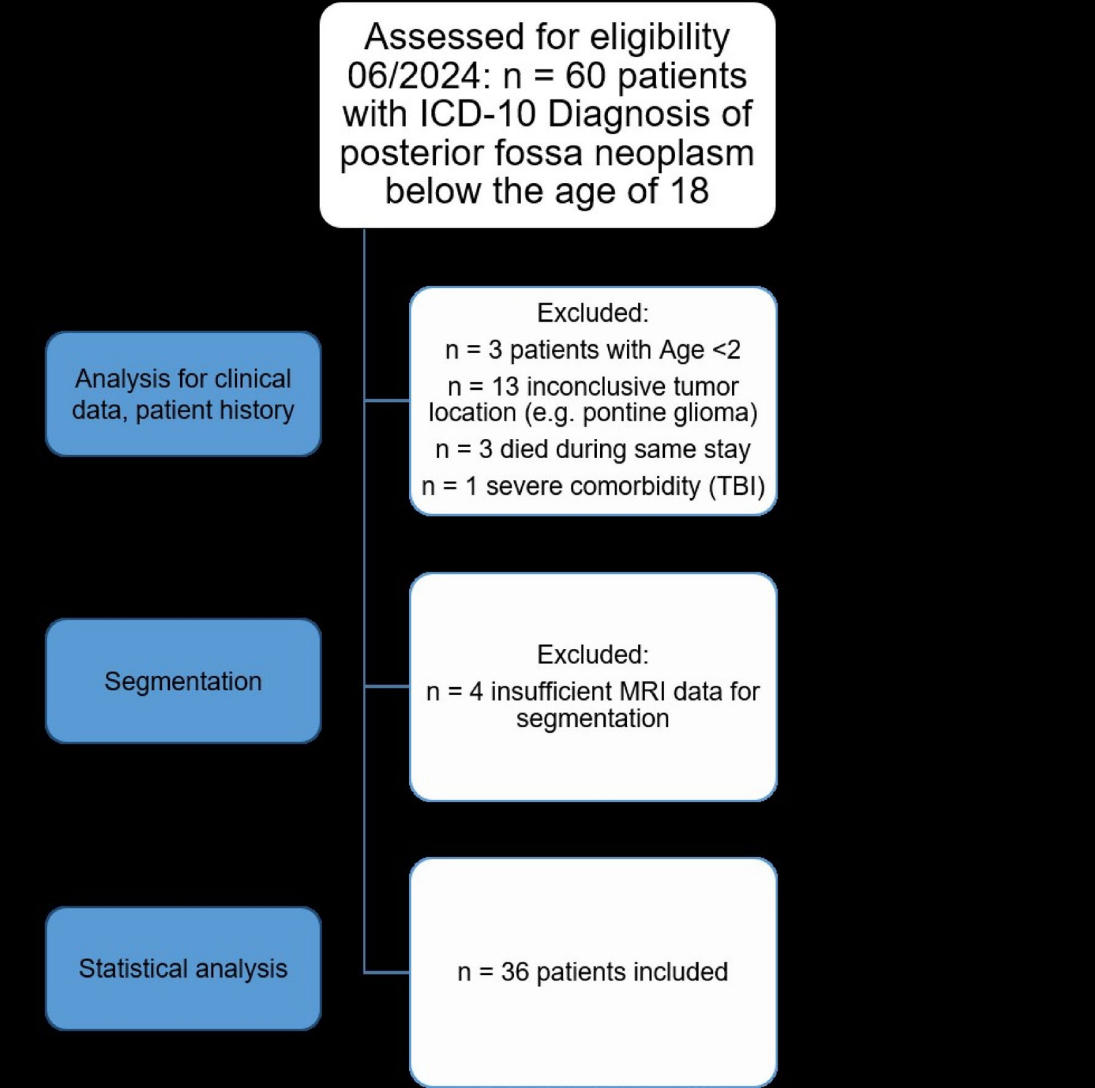
Flow chart for patient recruitment. TBI: traumatic brain injury. MRI: Magnetic resonance imaging.

60 patients undergoing surgery from 2012 to 2024 were eligible. *N* = 3 patients succumbed to the disease perioperatively due to surgical (*n* = 2) or non-surgical (*n* = 1) complications before shunt-dependency was determined. One child suffered from severe co-morbidity with traumatic brain injury. We proceeded to semiautomated segmentation, whereas *n* = 4 patients had incomplete MRI-datasets. After applying exclusion criteria, patient data for *n* = 36 were complete (s. Figure 2).

In addition to epidemiologic and baseline data of the patients (Table 1), potential radiomic and predescribed predictors of shunt dependency were compared between the group with downstream shunt placement or ETV (*n* = 7) and the children without the need for derivation (*n* = 29, Table 2).

**Table 1:** Patient characteristics and baseline data. ETV = endoscopic thirdventriculostomy. CSF = cerebrospinal fluid. HCP = hydrocephalus. mCPPRH = modified Canadian preoperative prediction rule for hydrocephalus. WHO = World health organisation. Cm³ = cubic centimeter. VBR = ventricle brain ratio. EVD = external ventricular drainage. VPS = ventriculoperitoneal shunt.

Characteristic	*N* = 36
Age (years)	8.5 (± 4.3)
Shunt-dependent	6
ETV postoperatively	1
Need for CSF diversion (Shunt or ETV)	7
Average time to implantation (days)	12.5 (± 3.5)
Papilledema	9
Transependymal edema	18
Severe HCP	12
mCPPRH high risk mCPPRH low risk mCPPRH average	1 35 1.72 (± 1.54)
Emergency resection	2
Extent of resection	
- Gross total	16
- Subtotal	18
- Biopsy	2
Entitity	
- Pilocytic Astrocytoma	16
- Medulloblastoma	13
- Ependymoma (anaplastic)	2 (1)
- Astrocytoma WHO 2	1
- Glioblastoma	1
- Ganglioglioma	1
- Choroid Plexus Papilloma	1
- Epidermoid Cyst	1
Radiomic Averages (cm³)	
Cerebrum	949.3 (± 130.0)
Ventricle volume	71.9 (± 61.5)
Brainstem	26.9 (± 4.8)
Posterior fossa	156.8 (± 33.9)
Whole brain	1204.91 (± 197.92)
VBR	0.055 (± 0.039)
Tumor volume	31.06 (± 22.81)
Cerebrum postop	949.2 (± 117.8)
Ventricle volume postop	53.4 (± 45.7)
Whole brain postop	1186.2 (± 173.4)
deltaVBR	0.82 (± 0.26)
HCP Management	
- ETV/EVD preoperatively	0 (0%)/5 (13.9%)
- EVD intraoperatively	7 (19.4%)
- Perioperative CSF diversion (all)	12 (33.3%)
- VPS	6 (16.7%)
- ETV	1 (2.8%)

Among the 36 patients eligible, 7 (19.4%) needed persistent CSF diversion (6 VPS; 1 ETV). 14 children underwent CSF diversion during acute treatment (7 pre- and 7 perioperatively), of which 5 (35.7%) required hydrocephalus treatment postoperatively. One child required hydrocephalus treatment after tumor resection and could be sucessfully diverted via ETV. In this case, “shunt-dependency” and requirement for hydrocephlus treatment are used synonymously. Perioperative CSF diversion was not significantly correlated to persistent shunt dependency (*p* = 0.08). The most common diagnoses were pilocytic astrocytoma (*n* = 16, 44.4%) and medulloblastoma (*n* = 13, 36.1%), followed by rarer entities. Brain volumina were assessed with an expectable variety in skull, brain and posterior fossa size. Larger tumors resulted in larger posterior fossa and larger whole brain volumes, consecutively, while brain stem volume was relatively constant. Mean ventricle volume decreased postoperatively by ~ 18 cm³.

**Table 2:** Comparative analysis between shunt-dependent and shunt-free patients in 1 year follow-up^1^.There was no significant difference in shunt-dependency between pilocytic Astrocytoma and medulloblastoma. All other entities were rare. VPS: ventriculoperitoneal shunt; HCP: hydrocephalus; mCPPRH: modified Canadian posterior Fossa prediction rule for hydrocephalus; VBR: ventricle brain ratio. DeltaVBR: relative difference in VBR pre/postoperatively; ETV: endoscopic third ventriculostomy; EVD: external ventricular drainage. CSF: cerebrospinal fluid. *considered to be significant with p < 0.05.

Characteristic	Shunt dependency (*n* = 7)		No VPS (*n* = 29)		*p*-values
	Mean/n		Mean/n		
Age	7.71 ± 5.15		8.79 ± 4.12		0.557
Papilledema	1		8		0.652
Transependymal edema	6		12		0.088
Severe HCP	3		10		0.499
mCPPRH high risk mCPPRH low risk mCPPRH average	1 6 1.17 ± 0.90		0 29 4.83 ± 3.63		0.356 0.429
Emergency resection	0		2		0.644
Extent of resection					0.119
- Gross total	1		15		
- Subtotal	6		12		
- Biopsy	0		2		
Entity^1^					
- Pilocytic Astrocytoma	3		13		0.374
- Medulloblastoma	4		9		
Volumetric parameters (cm³)					
Cerebrum	1030.56 ± 151.79		929.71 ± 118.93		0.065
Ventricle volume - above cut-off	128.14 ± 85.17 6		58.27 ± 46.77 9		0.075 0.011*
Brainstem	28.6 ± 6.46		26.54 ± 4.34		0.313
Posterior fossa	182.16 ± 24.51		150.68 ± 33.32		0.025*
Whole brain	1369.46 ± 220.48		1165.2 ± 173.63		0.012*
VBR - above cut-off	0.0896 ± 0.05205 6		0.0473 ± 0.03089 16		0.078 0.013*
Tumor volume	44.34 ± 20.43		27.74 ± 22.48		0.085
Cerebrum postoperatively	997.61 ± 106.32		937.46 ± 119.14		0.23
Ventricle volume postoperatively	81.18 ± 59.96		46.68 ± 40.13		0.073
Whole brain postop	1289.56 ± 136.44		1161.34 ± 174.03		0.079
deltaVBR - below cut-off	0.6642 ± 0.1986 1		0.859 ± 0.263 11		0.077 0.037*
HCP Management					
- ETV/EVD preoperatively	2		5		0.602
- EVD intraoperatively	3		4		0.1159
- Perioperative CSF diversion (all)	5		9		0.08

The proposed preoperative radiographic characteristics transependymal and papilledema were not significantly related to shunt dependency in our analyses (*p* = 0.088/*p* = 0.651). Regarding brain volumina, there was an overall significant correlation between larger volumina and shunt-dependency. Neither preoperative nor intraoperative EVD placement showed significant correlation to the necessitiy of VPS implantation. Furthermore, any kind of CSF diversion before resection was not significantly correlated to later VPS placement.

In volumetric data, several parameter were significantly correlated to later CSF diversion, such as whole-brain volume (*p* = 0.012) and posterior fossa volume (*p* = 0.013). Interestingly, tumor volume itself did not correlate with shunt dependency. In an automated pipeline, the determination of the posterior fossa is challenging, as the healthy anatomy is greatly altered. As to the scope of our study, we proceeded to examine VBR, deltaVBR for predictive value. Because of the simple radiografic identification and clearly significant correlation, we integrated ventricle size into our analysis.

We performed ROC analyses to determine the predictive value of the aformentioned parameters. Cut-off values were calculated by Youden’s Index. One sided t-test was performed to verify the results after dichotomization (s. Table 2).

**Fig. 3:**
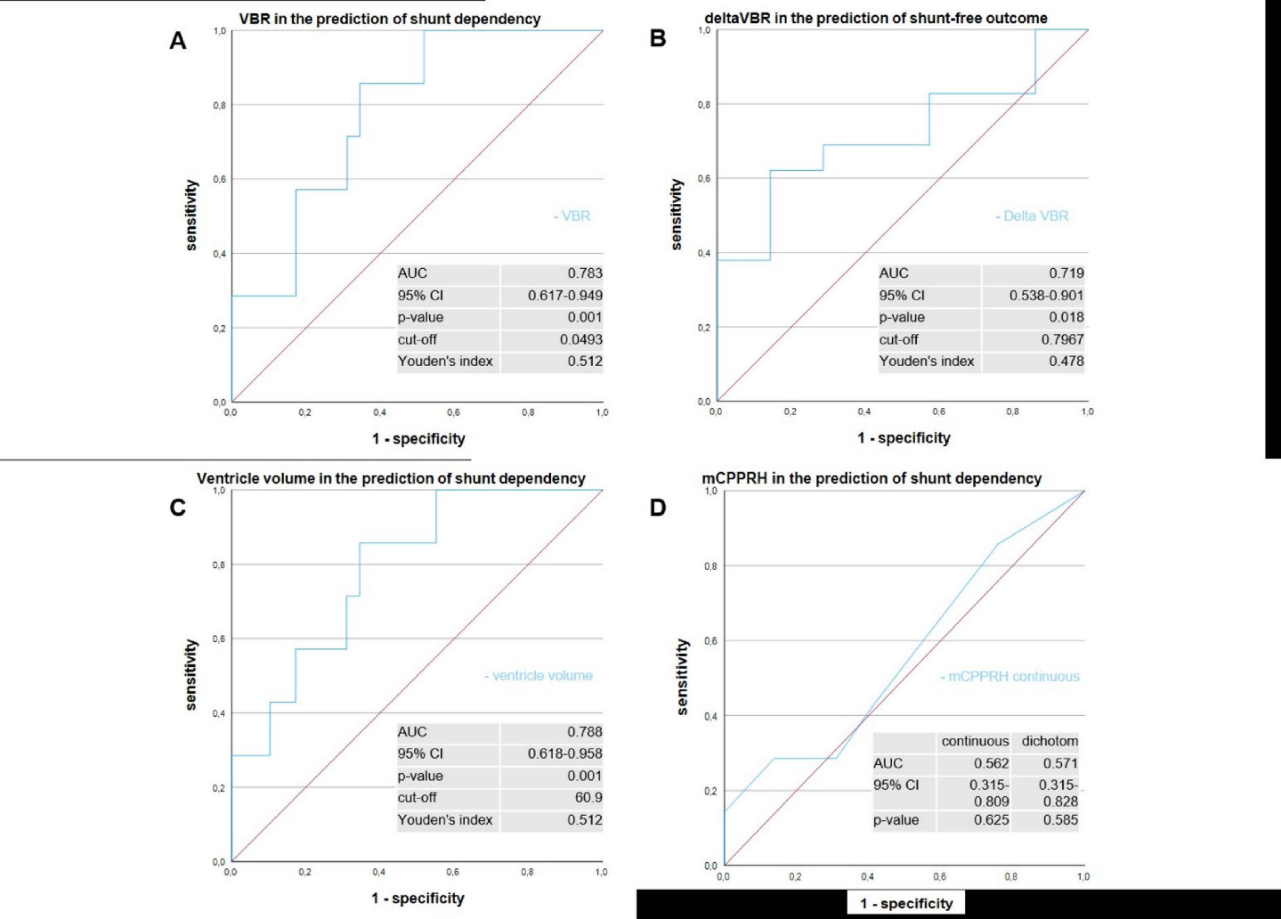
ROC-Analyses for prediction of shunt-free and shunt-dependent survival. **A**: ROC curve for VBR in the prediction shunt dependency. **B**: ROC curve for deltaVBR in the prediction of shunt-free survival. **C**: Ventricle volume as predictive marker of shunt dependency. **D**: mCPPRH (Modified Canadian Posterior Fossa Prediction Rule for Hydrocephalus) prediction as a continuous scale. Values are given for dichotomized AUc also (not shown). AUC: Area under the curve; CI: Confidence interval.

The ROC analyses for prediction of shunt-free and shunt-dependent survival are shown in Fig. 3. In ROC analyses for the predictive value of shunt implantation, VBR showed significant correlation with an AUC of 0.783 (*p* = 0.001). B: ROC-analysis for the prediction of shunt-free survival compared to the overall need for CSF diversion showed significant correlation between deltaVBR and shunt-free follow-up (AUC = 0.719, *p* = 0.018). A decrease in ventricle-to-brain size after surgery was correlated to shunt-free survival. Ventricle volume alone as predictive marker of shunt-dependency prooved to be significantly correlated to shunt-dependency (AUC = 0.788, *p* = 0.001, cut-off 60.9 ml). In our cohort, mCPPRH (Modified Canadian Posterior Fossa Prediction Rule for Hydrocephalus) had no value in VPS prediction (AUC 0.562/0.571).

## Discussion

### VBR as predictor of shunt-dependency

We chose the relation of ventricle to whole brain volume as a potential marker based on these major assumptions:

1. Increased ventricle volume is a direct consequence of CSF occlusion^15^.
2. As the cerebral capacity to adapt to occlusion depends on time, larger ventricles may only occur in a relatively slow process of persistent, increasing occlusion.
3. Whole brain volume, e.g. skull capacity, should remain relatively constant pre- and postoperatively, as the skull is rather rigid^16^.
4. Ventricle size increases related to age, but not anywhere near the changes it underlies in hydrocephalus^17^. A ratio between ventricle and whole brain size is therefore not significantly influenced by the child’s age.

In our first attempt to find radiomic features for shunt prediction, the relation of ventricle volume to brain size yielded significant correlation (*p* = 0.008). In ROC analysis, it showed high predictive value for shunt dependency. However, when applied to our small cohort, confidence intervals indicated low reliability and indicated a lack of significance (OR 7.385, CI = 0.786–69.361). This is a direct result of small sample size and few events (VPS implantation), but could also indicate high variability in primary data (segmented volumes) or model instability. This is especially likely due to the variety of risk factors that can make CSF diversion necessary and the therefor multicorrelated model. In further statistical analysis, the change in this ratio from pre- to postoperatively showed relative negative predictive value for CSF circulation restoration (OR 9.818, CI = 1.2–108.293). While these results indicate statistical significance, we again face large confidence intervals as a result of a small sample size with few events. Volume variability remains high, with a large impact on proposed quota (VBR and deltaVBR). We therefore conclude, that a decrease in ventricle size in relation to skull capacity postoperatively could be an indicator for a normalized CSF circulation, thus making shunting obsolete. These findings are nonetheless confounded by MRI timing and EVD management, such as amount of CSF drainage and operating procedure of EVD weaning. Logically, presurgical parameters might be of higher predictive value.

### Ventricle size and shunt dependency in chronic pediatric hydrocephalus

The strongest correlation to a rather easy-to-access radiomic feature turned out to be ventricle volume. Being the main defining criteria for hydrocephalus as such^18^, this may come as no surprise. When comparing pre- and postoperative measurements and assessing their predictive value, we could nonetheless show, that shunt dependency might as well be determined pre-resection – regardless of perioperative CSF diversion. This might support the hypothesis, that CSF circulation has mostly been impaired beyond repair in children with the need for ETV or EVD perioperatively^6^. In our opinion, the need for perioperative CSF diversion therefore is and will be a mainly clinical one. As current applications make semiautomated ventricle volumetry quick and reliable^19^; ventricle volume alone can be easily assessed to stratify the risk for persistent shunt dependency and can keep practitioners alerted in that regard.

The most interesting finding of ventricle volume analysis was, however, that the cutoff for shunt dependency was very high at 60 ml. This is well above the 95th percentile of normal distribution of ventricle volume in children, which is above 20 ml at age 2 and surpasses 30 ml only at age 16^20^. In a series of 617 children, not a single otherwise healthy child surpassed 50 ml. While ventricle size can surpass 60 ml in cases of unspecific ventriculomegaly, this occasion seems to be quite rare. In this cohort, a more likely pathophysiological mechanism is the proposed chronic adapation to reduced CSF outflow via the fourth ventricle. Therefore, the necessity for CSF diversion seems to differ greatly in chronic pediatric hydrocephalus, compared to acute onset.

### Radiomic score

In search of a potential predictor using solely radiomic features we combined ventricle size beyond cut-off with transependymal edema, another highly correlated feature. It has been described before as predictive and has been used in the mCPPRH Score. We developed a grading system diverting patients into the risk groups mentioned above, showing a reliable odd’s ratio of 9.583 (CI = 1.477–62.169) in shunt-dependency prediction for high-risk patients. Both ventricle size and transependymal edema are easily accessible, making the estimation for HCP severity and possible peristence feasible in an emergency setting.

### Retrospective mCPPRH score analysis in our cohort

After evaluation of the predicitve markers mentioned above, we analyzed our cohort based on the mCPPRH Score. Of 36 patients, only one patient was stratified into the “high risk” group for shunt dependency, while 7 patients needed persistent CSF diversion This does not allow stratification in comparable groups, thus resulting in a low predictive value. In the course of 16 years, the score would have had minor value in shunt prediction in our facility. This aligns with other studies focusing on its validation^4,13^.

### Limitations

Definition of shunt-dependency: The fact that the patient has received ventriculo-peritoneal shunt implantation is based on clinical decision making and lastly subjected to the neurosurgeons discretion. Furthermore, the decision for preoperative CSF diversion is made in an acute setting by the attending physician. Some children are CSF-diverted externally with a vital indication and referred to our hospital afterwards. This decision clearly affects further HCP development and remains a sginificant confounder. It is an important hint to, but not a proof of shunt-dependency, especially during recovery of the patient, which might be accelerated by shunt-placement. The small sample size and the retrospective character of the study clearly limitate statistical power. Per design, this study can not clarify causality, while pathophysiological theoretization is not the aim of this study. It therefore remains a proof-of-concept and proposal, which needs further validation. Especially the retrospective nature, relatively small sample size, and unknown inter-rater reliability.

## Conclusions

Ventricle-brain ratio is a potential predictor for the risk of the necessity of CSF diversion, while its reliability remains unproven. The cut-off in ventricle volume for shunt dependency in chronic obstructive pediatric hydrocephalus (60 ml) is nowhere near the 95th percentile of healthy controls. In our cohort, radiomic predictors were more relatively more reliable than hydrocephalus categorization, modified Canadian Preoperative Prediction Rule for Hydrocephalus (mCPPRH) or transependymal edema alone. VBR pre- and deltaVBR are potentially useful tools to predict the need for shunting in pediatric posterior fossa tumor patients. Their predictive value has yet to be validated in larger cohorts. The decision for pre- or intraoperative CSF diversion showed no correlation and no influence on persistent hydrocephalus. The clinical presentation of postoperative shunt dependency itself might rather be already determined preoperatively, manifested in an absurdely large ventricular system. If we assume shunt dependency a result of chronically impaired CSF circulation, it shall be uninfluenced by perioperative HCP management. As there are always exceptions from proposed rules and the patients’ symptoms should be our guidance, ETV/EVD placement as well as shunt implantation remains a clinical decision. Preoperative volumetry can enable us to estimate the likelihood of postoperative shunt-dependency, therefore increasing alertness in postoperative surveillance.

## Data Availability

The datasets used and/or analysed during the current study available from the corresponding author on reasonable request.

## Declarations

### Previous Presentations

A short talk on the data was held at *Sektionstagung der DGNC*, Würzburg, Germany, 21st of Spetember 2024.
